# Celiotomy

**Published:** 1904-06

**Authors:** 


### Celiotomy.

In various communications to medical journals during the last few years, we have noticed this term used to describe the procedure now generally spoken of as abdominal section. The old-fashioned term "laparotomy" is sanctified by usage, and is generally understood, though it labors under the etymological disadvantage of signifying "loin-cutting," and cannot therefore be defended, philologically, as a designation for incision through the anterior abdominal parietes. If, however, we are to change it, let us at least adopt a term that is beyond cavil; for us celiotomy seems open to grave objection. We believe the originator of the word was Mr. Bland-Sutton, who is quite a good enough biologist to know the peritoneal cavity is not the only representative of the celom in the body, and to use a term that is based upon the assumption that it is, is to court misunderstanding by unnecessary ambiguity. Any operation in which the pleura or pericardium was incised could be equally accurately described as a celiotomy, for both these cavities represent in the adult the space formed by the mesoblastic splitting in the embryo. We protest, therefore, against the adoption of a term which violates convention without ensuring accuracy. We are no opponents of reform in nosology, and we should gladly welcome a good substitute for laparatomy, but we would rather "bear those ills we have than fly to others," equally grievous, and less generally understood.—*Med. Press and Circular.*

